# Distinct roles for the anterior temporal lobe and angular gyrus in the spatiotemporal cortical semantic network

**DOI:** 10.1093/cercor/bhab501

**Published:** 2022-01-30

**Authors:** Seyedeh-Rezvan Farahibozorg, Richard N Henson, Anna M Woollams, Olaf Hauk

**Affiliations:** MRC Cognition and Brain Sciences Unit, University of Cambridge, Cambridge, CB2 7EF, UK; Wellcome Centre for Integrative Neuroimaging, Nuffield Department of Clinical Neurosciences, University of Oxford, Oxford, OX3 9DU, UK; MRC Cognition and Brain Sciences Unit, University of Cambridge, Cambridge, CB2 7EF, UK; Department of Psychiatry, University of Cambridge, Cambridge, CB2 0SZ, UK; Neuroscience and Aphasia Research Unit, School of Biological Sciences, University of Manchester, Manchester, M13 9PL, UK; MRC Cognition and Brain Sciences Unit, University of Cambridge, Cambridge, CB2 7EF, UK

**Keywords:** connectivity modeling, dynamic causal modeling, EEG/MEG source estimation, semantic network, spatiotemporal dynamics

## Abstract

Semantic knowledge is supported by numerous brain regions, but the spatiotemporal configuration of the network that links these areas remains an open question. The hub-and-spokes model posits that a central semantic hub coordinates this network. In this study, we explored distinct aspects that define a semantic hub, as reflected in the spatiotemporal modulation of neural activity and connectivity by semantic variables, from the earliest stages of semantic processing. We used source-reconstructed electro/magnetoencephalography, and investigated the concreteness contrast across three tasks. In a whole-cortex analysis, the left anterior temporal lobe (ATL) was the only area that showed modulation of evoked brain activity from 100 ms post-stimulus. Furthermore, using Dynamic Causal Modeling of the evoked responses, we investigated effective connectivity amongst the candidate semantic hub regions, that is, left ATL, supramarginal/angular gyrus (SMG/AG), middle temporal gyrus, and inferior frontal gyrus. We found that models with a single semantic hub showed the highest Bayesian evidence, and the hub region was found to change from ATL (within 250 ms) to SMG/AG (within 450 ms) over time. Our results support a single semantic hub view, with ATL showing sustained modulation of neural activity by semantics, and both ATL and AG underlying connectivity depending on the stage of semantic processing.

## Introduction

When we see a word, how do we understand its meaning? What areas in the brain are involved and how do these areas connect over time to form the neuronal networks that support retrieval of semantic knowledge? Ample evidence, predominantly based on neuropsychology and functional magnetic resonance imaging (fMRI) (Binder et al. 2009; Lambon Ralph et al. 2016), points to the involvement of a large number of brain regions in semantic processing, particularly in the left hemispheric temporal, parietal, and frontal regions (Binder and Desai 2011). However, the precise role of each area within this network, and importantly the spatial and temporal configuration of the network that binds these areas together, remains an open question (Pulvermüller 2013; Hauk 2016; Rogers et al. 2021). In order to delineate this network further, we focus on the first few hundred milliseconds of word processing, which have already been shown to include distinct aspects of lexico-semantic processing (Lau et al. 2008, 2013; Hauk et al. 2012; Westerlund and Pylkkänen 2014; Pylkkänen 2016). Therefore, we here aim to trace the fast, transient states of brain activity and connectivity across these early stages, which are not resolvable with fMRI.

One of the most prominent models of semantic processing is the hub-and-spokes model (Patterson et al. 2007), which proposes that a single hub region in the anterior temporal lobe (ATL) binds the semantic network together (Rogers et al. 2004; Lambon Ralph et al. 2016). Strong evidence for this model has come from computational modeling (Rogers et al. 2004; Chen et al. 2017; Hoffman et al. 2018), neuroimaging research (Lau et al. 2013; Binney et al. 2016; Jackson et al. 2016; Lambon Ralph et al. 2016), and from studies on semantic dementia, a type of neurodegenerative brain disorder that is associated with damage to the ATLs and a profound loss of semantic knowledge (Snowden et al. 1989, 2017; Rogers et al. 2004; Patterson et al. 2007). A prominent alternative model is the theory of convergence zones (Barsalou 2009; Meyer and Damasio 2009; Martin 2016), where multiple heteromodal regions, for example, in the posterior inferior parietal lobe (IPL; e.g., angular gyrus, AG), middle temporal gyrus (MTG) and inferior frontal gyrus (IFG), are suggested to play equally central roles. Meta-analytic neuroimaging evidence based on fMRI and positron emission tomography (Binder et al. 2009; Pulvermüller 2013), as well as neurocomputational modeling of word learning (Tomasello et al. 2017) supports the existence of multiple semantic convergence zones.

The hub-and-spokes model makes predictions about the role of the semantic hub that can be used to explicitly test the model against other frameworks. More specifically, the hub is proposed to act as a mediator layer of the network that receives input from the sensory regions, for example, visual cortex for the visually presented words, and mediates concept retrieval through establishing multiple connections to other key semantic areas (Rogers et al. 2004; Patterson et al. 2007; Chen et al. 2017). As a result, we hypothesized that both the neural activity within the hub area and its connectivity with the rest of the semantic network should be modulated by semantic variables as of the earliest stages of semantic processing. We tested these two key hypotheses by leveraging the fine spatiotemporal resolution of source-reconstructed electroencephalography (EEG) and magnetoencephalography (MEG) to trace modulation of brain activity and effective connectivity (via Dynamic Causal Modeling; DCM) over time.

For this purpose, we examined the contrast of visually presented concrete and abstract words across three tasks from two experiments. The concreteness contrast was chosen, as in a number of previous neuroimaging studies (Fiebach and Friederici 2004; Binder et al. 2005; Dhond et al. 2007; Hoffman 2016; Gao et al. 2019), because concrete and abstract words differ with respect to their general semantic processing demands and their reliance on distributed semantic areas (Binder et al. 2005; Dhond et al. 2007). More specifically, the representations of concrete words are thought to be mediated through interactions between the heteromodal semantic system and the brain regions that underlie nonverbal imagery or provide context availability (Jessen et al. 2000; Sadoski et al. 2006; Paivio 1990; Hoffman 2016; Taylor et al. 2019). Abstract words, on the other hand, rely more strongly on symbolic semantic representations in the heteromodal regions, and thus can be expected to place more demand on putative hub areas (Jessen et al. 2000; Sadoski et al. 2006; Paivio 1990; Hoffman 2016; Taylor et al. 2019). Furthermore, superior comprehension of concrete than abstract concepts is a well-established feature of semantic dementia (Reilly et al. 2006; Jefferies et al. 2009; Loiselle et al. 2012; Hoffman et al. 2013). Therefore, this “concreteness contrast” can be expected to modulate activation in and connectivity profiles of the putative hub(s).

In order to identify core semantic effects of concreteness that are minimally confounded by specific task demands, we sought results based on accumulated evidence across three tasks: lexical decision, concreteness decision, and semantic target detection. Previous research on concreteness effects has typically used a single task, for example, concreteness judgment, or lexical decision. Concreteness decisions have the benefit of explicitly forcing participants to think about the dimension of interest, but confound concrete versus abstract conditions with different responses (e.g., “yes” vs. “no”). Lexical decision removes the confound of different response types (since both conditions require a “yes” response), but can potentially be performed without deep semantic processing. In addition to these two most commonly used tasks, we ran a third, semantic target detection task, where participants silently read stimuli and only responded to rare targets that were not of interest, thus requiring semantic processing without any response to the non-targets of interest. This allowed us to identify the commonalities in concreteness effects across tasks.

Our analyses focussed on two aspects of our data, namely evoked brain responses and effective connectivity. First, we conducted vertex-wise general linear modeling (GLM) of the evoked brain activity over multiple time windows spanning 50–450 ms post-stimulus. Using whole-cortex cluster-based permutations, we identified the spatiotemporal clusters whose activity was modulated by the main effect of concreteness across tasks. Second, using DCM of evoked responses (David et al. 2006), we tested for the presence of a central hub within the heteromodal semantic subnetwork that links sensory inputs to different nodes of this network in two latency ranges (within 250 and 450 ms). For this purpose, we constructed a hierarchy of model comparisons comprising two levels, and asked: 1) are models with a single connectivity hub preferred over models with no hubs, and 2) in the preferred models, do the areas that function as a hub change across the course of semantic retrieval? In order to obviate task- and experiment-specific effects, here we identified the winning family of DCM models in each level of hierarchy based on accumulated Bayesian evidence across the three tasks.

## Materials and Methods

We present data from two separate EEG/MEG experiments, all based on visual word recognition paradigms, and the contrast of concrete and abstract words. The first experiment used a concreteness decision task and the second a lexical decision as well as a semantic target detection task.

### Experiment 1—Concreteness Decision

#### Participants

Twenty healthy native English speakers participated in the study, but three participants were removed due to excessive movement artifacts or measurement error. Hence 17 participants (age 27 ± 6 years, 12 female) entered the final analysis. A mean handedness laterality quotient of 82 (min 41, max 100) was obtained from a reduced version of the Oldfield handedness inventory (Oldfield 1971). All participants had normal or corrected-to-normal vision with no reported history of neurological disorders or dyslexia. The experiment was approved by the Cambridge Psychology Research Ethics Committee, it was undertaken with the understanding and written consent of each participant, and volunteers were paid for their time and effort.

#### Stimuli

Participants were presented with 184 abstract and concrete words (92 per category), matched for a number of psycholinguistic variables including Kucera–Francis and CELEX word frequencies, familiarity, concreteness, and imageability ratings as well as the number of letters/phonemes/syllables (for details, see Table 1). Concreteness ratings were taken from the MRC Psycholinguistic Database (Coltheart 1981) and CELEX frequency, orthographic neighborhood, bigram and trigram frequencies were taken from the MCWord Database (Medler and Binder 2005). The two categories differed significantly on concreteness (*t*s > 19.3575, *P*s < 0.0005) as indicated by independent samples *t*-tests, but not with respect to the other aforementioned variables. As a post-hoc test (conducted after data collection), we also calculated morphological complexity for the stimuli based on the MorphoLEX database (Sánchez-Gutiérrez et al. 2018). Of the 184 stimuli, 149 were monomorphemic (82 concrete, 67 abstract), 34 were bimorphemic (10 concrete, 24 abstract), and 1 was trimorphemic (0 concrete, 1 abstract). Of the bimorphemic and trimorphemic stimuli, 3 were transparent compounds (all three concrete), 1 was opaque compound (1 concrete), and the remaining 31 were prefixed or suffixed words.

**Table 1:** Psycholinguistic properties of stimuli in Experiment 1 for relevant stimulus dimensions

	Concrete (average ± std)	Abstract (average ± std)
Number of Letters	5.95 ± 0.8	5.95 ± 0.8
CELEX Frequency	11.8 ± 14.9	15.28 ± 15.4
Orth Neighborhood	1.64 ± 2.1	1.38 ± 2.2
Number of Syllables	2.0 ± 0.6	2.1 ± 0.8
Bigram Frequency	19705.18 ± 8020.2	21530.53 ± 11210.2
Trigram Frequency	1704.99 ± 1339.9	2200.4 ± 3304.8
Concreteness Rating	590.0 ± 26.0	303.0 ± 39.0

std: standard deviation.

#### Procedure and Task

Single-word stimuli appeared as 28-point Arial font in white on a black screen within a visual angle of 4 degrees in a slightly dimmed and acoustically shielded MEG chamber at the MRC Cognition and Brain Sciences Unit, University of Cambridge. Duration of stimulus presentation was 150 ms, with an average SOA of 2400 ms (uniformly jittered between 2150 and 2650 ms). Participants performed a concreteness decision task, by performing button presses with their right hand, using index and middle fingers to distinguish concrete and abstract words. Short breaks were included after approximately every 50 trials. Participants were given a few minutes of practice prior to the experiment, using different stimuli, until they felt comfortable with the task. The first two trials (filler items) after each break and at the beginning of each block were not included in the analysis.

### Experiment 2—Semantic Target Detection and Lexical Decision

This experiment included two tasks, a semantic target detection and a lexical decision task. Similar to the previous experiment, we investigated the contrast of concrete and abstract words using a visual word recognition paradigm. Stimuli for this experiment were selected using a separate word rating study, with a separate group of participants who did not have any overlaps with participants of the EEG/MEG part of the study (for details of word rating, see Supplementary Material 1).

#### Participants

Twenty-six healthy native adult English speakers (age 18–40 years) participated in the study, of which two were removed due to problems with structural MRI scans, three were removed due to inadequate behavioral response accuracies, and three were removed due to excessive movement artifacts. Hence, 18 participants (mean age 27.00 ± 5.13 years, 12 female) entered the final analysis. A handedness laterality quotient of 89.84 ± 0.2 was obtained from a reduced version of the Oldfield handedness inventory (Oldfield 1971). All participants had normal or corrected-to-normal vision with no reported history of neurological disorders or dyslexia. The experiment was approved by the Cambridge Psychology Research Ethics Committee, it was undertaken with the understanding and written consent of each participant, and volunteers were paid for their time and effort.

#### Stimuli

Participants were presented with 150 concrete words grouped into three categories of visual, auditory and hand-action words (50 words per category) as well as 50 abstract words. For this analysis, we selected 50 concrete words to match the abstract words with respect to a number of psycholinguistic variables (details presented in Table 2). Concreteness ratings were obtained based on the complementary word rating study (see Supplementary Materials 1) and CELEX frequency, orthographic neighborhood, bigram, and trigram frequencies were taken from the MCWord Database (Medler and Binder 2005). Additional filler pseudowords and words were also included in the experiment, which are not of interest in this study. Similar to Experiment 1, we ran a post-hoc test (conducted after data collection) to calculate morphological complexity for the stimuli based on the MorphoLEX database (Sánchez-Gutiérrez et al. 2018). Of the 100 stimuli, 77 were monomorphemic (46 concrete, 31 abstract), 18 were bimorphemic (4 concrete, 14 abstract), and 5 were trimorphemic (0 concrete, 5 abstract). Of the bimorphemic and trimorphemic stimuli, 4 were transparent compounds (2 concrete, 2 abstract), and the remaining 19 were prefixed or suffixed words.

**Table 2:** Psycholinguistic properties of stimuli in Experiment 2 for relevant stimulus dimensions

	Concrete (average ± std)	Abstract (average ± std)
Number of Letters	6.2 ± 1.21	6.44 ± 1.51
CELEX Frequency	17.43 ± 23.87	18.03 ± 21.19
Orth Neighborhood	1.62 ± 2.44	2.12 ± 3.61
Number of Syllables	1.8 ± 0.64	2.22 ± 1
Bigram Frequency	21313.27 ± 11343.31	20652.47 ± 7988.63
Trigram Frequency	2167.46 ± 3420.88	2022.06 ± 1282.76
Concreteness Rating	5.61 ± 0.96	2.68 ± 0.48

Some previous studies have reported ATL modulation by transparent compounds, but no modulation by opaque compounds (Brooks and De Garcia 2015), suffixed, or pseudo-suffixed words (Flick et al. 2018) compared with monomorphemic stimuli. Interestingly, Flick et al. (2018) argued that ATL sensitivity to transparent compounds is potentially linked to its sensitivity in semantic composition (e.g., Pylkkänen 2019) and its role for combining concepts. Considering that non-monomorphemic stimuli in both our experiments are predominantly prefixed/suffixed words, with only 3 of 184 transparent compounds (all concrete) in Experiment 1, and 4 of 100 transparent compounds (2 concrete, 2 abstract) in Experiment 2, we expect the confounding effect of morphological complexity on semantic processing, especially in ATL, to be minimal in our study.

#### Procedure and Task

The EEG/MEG experiment consisted of four randomized blocks, and lasted approximately 90 minutes. We included short breaks every 3 minutes and longer breaks between the blocks. Duration of stimulus presentation was 150 ms, with an average SOA of 2400 ms (uniformly jittered between 2150 and 2650 ms). Stimuli appeared as 30-point Arial font in black on a gray screen within a visual angle of 4 degrees in a slightly dimmed and acoustically shielded MEG chamber. Three blocks of the experiment consisted of semantic target detection tasks and the fourth block was a lexical decision task, where half of the subjects performed the lexical decision block before semantic blocks and half of them afterwards. Details of these blocks were as follows:

1. Semantic target detection blocks:In each block, participants were presented with concrete and the abstract words, as well as the filler items (overall 300 stimuli), in addition to 30 targets. They were asked to quietly read the strings of letters as they appeared on the screen and make button press responses with the middle finger of their left hand whenever a target appeared on the screen. Each block had different targets which were selected from three groups of “non-citrus fruits”, “something edible with distinctive odor”, and “food that contains milk, flour, or egg”. These semantic target categories were chosen in order to require access to specific semantic information, and did not require an explicit distinction between concrete and abstract meanings. Blocks were presented in a random order and data obtained from the three blocks were pooled in the later EEG/MEG analyses so as to obviate possible question-specific effects.
2. Lexical decision task:In addition to the target detection task, participants also performed a lexical decision task with all the words and pseudowords (and additional 150 filler pseudowords compared to semantic blocks in order to match the overall number of words to acquire response balance). Participants were asked to make button press responses with the index and ring fingers of their left hand indicating whether or not “the following string of letters refers to a meaningful word”.

### E‌EG/MEG Data Acquisition

MEG data for both experiments were acquired in a magnetically shielded room using a Neuromag Vectorview system (Elekta AB, Stockholm, Sweden), with 204 planar gradiometers and 102 magnetometers (i.e., 306 channels overall). EEG data were collected concurrently using a 70-electrode EEG cap (EasyCap GmbH, Herrsching, Germany). EEG reference and ground electrodes were attached to the nose and left cheek, respectively. The electrooculogram (EOG) was recorded by placing electrodes above and below the left eye (vertical EOG) and at the outer canthi (horizontal EOG). Data were acquired with a sampling rate of 1000 Hz and a band pass filter of 0.03–330 Hz. Prior to the MEG recording, the positions of 5 head position indicator (HPI) coils attached to the EEG cap, 3 anatomical landmark points (two ears and nose) as well as approximately 50–100 additional points covering the whole EEG cap were digitized using a 3Space Isotrak II System (Polhemus, Colchester, Vermont, USA) for later coregistration with MRI data.

#### Rationale for Using Concurrent EEG/MEG

The choice of concurrent EEG and MEG in this study was made to maximize spatial resolution. EEG/MEG source estimation is an ill-posed problem, where sources of brain activity can only be estimated using mathematical inverse solutions but cannot be exactly determined. Previous studies have shown that using combined MEG and EEG results in higher spatial resolution (Sharon et al. 2007; Molins et al. 2008; Henson et al. 2009; Hauk et al. 2019). This will specifically allow us to obtain improved source-localized signals from the candidate hubs of interest. In particular, due to issues pertaining to sensor coverage, MEG and EEG have been shown to yield less accurate signals from frontal and temporo-parietal brain regions, respectively (Coquelet et al. 2020; Hill et al. 2020). Therefore, using both modalities is expected to allow accessing their complementary strengths for obtaining signals from the candidate hubs in the frontal and parieto-temporal regions. While this combination can arguably allow us to detect any interesting effects more accurately, it remains to be determined in the future studies whether and how the results will differ if a single modality is used.

### E‌EG/MEG Data Processing in Sensor Space

The first step of data preprocessing included applying signal-space separation implemented in the Maxfilter software (version 2.0) of Elekta Neuromag to the raw MEG data in order to remove noise from sources distant to the sensor array (Taulu and Kajola 2005). The Maxfilter software also involved movement compensation and bad channel interpolation for MEG data. All the next analysis steps (except DCM and general linear modeling) were performed in the MNE-Python software package (http://martinos.org/mne/stable/index.html) (Gramfort et al. 2013, 2014). Raw data were visually inspected for each participant, and consistently bad EEG channels were marked and interpolated. Data were then FIR band-pass filtered between 1 and 48 Hz, using forward–backward filtering to achieve zero phase delay. Independent component analysis (ICA) was applied to the filtered data in order to remove eye movement and heart artifacts. We used the FastICA algorithm (Hyvärinen and Oja 2000) as included in scikit-learn python package (Pedregosa et al. 2011) and implemented in MNE-Python meeg-preprocessing package (with minor manual changes to achieve a better artifact rejection for some participants). After ICA, data were divided into epochs, between -500 ms and 700 ms for Experiment 1 and between −300 and 600 for Experiment 2, around the word onsets. Epochs were rejected if peak-to-peak amplitudes were higher than the following thresholds, based on previous norms: 120 μV in the EEG (except for two cases where we increased the threshold to 150 μV, because high rejection rates could be identified as due to excessive alpha activity despite good behavioral performance), 2500 fT in magnetometers, 1000 fT/cm for gradiometers. Trials with incorrect responses were also excluded from further analysis.

In this study, we only conducted statistical comparisons between concrete and abstract words in source space (details in the following sections). However, to provide a complete presentation of the data, in Supplementary Material 2 we also show sensor-space group averages per task and separately for each sensor type (EEG, MEG gradiometers, and magnetometers). Please refer to (Supplementary Figures 1 and 2).

### Forward Model and Inverse Solution

We used MNE and MNE-Python software packages to compute forward and inverse models, respectively. The forward model was computed based on a boundary element model (BEM) of the head derived from structural MR images for each participant. EEG/MEG sensor configurations and MR images were coregistered based on the aforementioned digitization points. Structural MR images were processed using the automated segmentation algorithms in FreeSurfer software (version 5.3; http://surfer.nmr.mgh.harvard.edu/) in order to obtain the reconstructed scalp surface (Dale et al. 1999; Fischl et al. 1999). The result of the FreeSurfer segmentation was processed further using MNE software package (version 2.7.3) and the original triangulated cortical surface, which included more than 160 000 vertices per hemisphere, was down-sampled to a tessellated grid in which the average edge of each triangle was approximately 2.5 mm (Segonne et al. 2004). A three-layer BEM containing 5120 triangles per layer was created for EEG and MEG from scalp, outer skull surface and inner skull surface, respectively. The noise covariance matrices for each dataset were computed and regularized using diagonal loading with a regularization factor of 0.1 for all channel types. Baseline intervals of 500 ms duration pre-stimulus were used for noise covariance estimation. The resulting regularized noise covariance matrix was used to assemble the inverse operator for each participant using L2 minimum-norm estimation (L2 MNE) with a loose orientation constraint value of 0.2 and without depth weighting.

### Source-Reconstructed Evoked Analysis

The inverse operator described in the previous section was next used for source reconstruction. After removing bad trials according to aforementioned criteria in Section EEG/MEG Data Processing in Sensor Space, the number of epochs was equalized between concrete and abstract words by matching the time of trial presentation. Trials for each condition were averaged in sensor space in order to yield an evoked response per participant and condition, which were then projected onto the source space using L2 MNE. We used MNE-Python’s default Signal to Noise Ratio (SNR) = 3.0 for regularization of the inverse operator for evoked responses. In order to obtain an unsigned evoked response, we computed activity with the loose orientation constraint. Afterwards, the individual participant results were morphed to the standard average brain (fsaverage5) in Freesurfer software, yielding time courses of activity for 20 484 vertices for each participant and condition. Source-estimated time courses were then averaged in four time windows from 50 to 450 ms with 100 ms increments for statistical analysis. These four time windows allowed us to cover the early stages of semantic processing up to, and including, the N400 time window, particularly the critical stages reported previously, e.g., up to 150 (Moseley et al. 2013; Teige et al. 2018); 150–250 ms (Bemis and Pylkkänen 2011; Hauk et al. 2012); 250–350 ms (Dhond et al. 2007); 350–450 ms (Lau et al. 2013).

### Whole-Cortex Statistical Inference

#### General Linear Model

**Figure 1:**
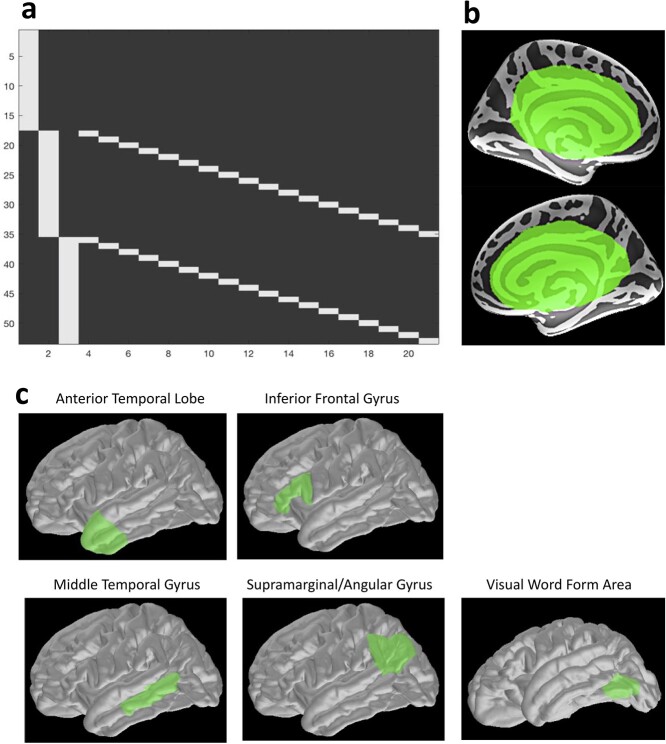
Specifics of GLM and DCM: (*a*) design matrix of whole-cortex vertex-wise GLM analysis; (*b*) vertices excluded from whole-cortex statistical analyses: green labels are defined manually (informed by the previous studies; e.g., Liu et al. 1998; Hauk et al. 2011; Farahibozorg et al. 2018) and mark deeper brain areas that were removed from the whole-cortex statistical analysis due to the limited spatial resolution of the EEG/MEG source localization; (*c*) five regions of interest in DCM models include anterior temporal lobe, inferior frontal gyrus, middle temporal gyrus, supramarginal/angular gyrus, and visual word form area in the left fusiform gyrus.

In order to find modulation of cortical evoked activity by semantic variables, we used a whole-cortex statistical analysis. For this purpose, we applied general linear modeling (GLM) and obtained *t*-statistics to summarize the main effect of concreteness across the three tasks in every brain vertex, as well as each of the four time windows of interest. The design matrix for the model is shown in Figure 1*a*, where columns 1–3 encode each task and columns 4–21 encode repeated subjects (among semantic target detection and lexical decision tasks from Experiment 2). It is worth noting that because the two experiments comprise different participants, this GLM design takes both within- and between-group variance into account. The GLM is conducted using *GeneralLinearModel* function in NIPY, and under the assumption of white Gaussian residuals.

#### Cluster-Based Permutations on GLM Results: Correction for Multiple Comparisons

For whole-cortex analyses, as we conduct here, it is essential to correct the results for multiple comparisons. We used spatiotemporal cluster-based permutations (Maris and Oostenveld 2007) in order to correct across both vertex and time dimensions and obtain significant clusters. For this purpose, we first thresholded the *t*-maps at a *t*-value corresponding to *P*-value = 0.05 (two-tailed). Cluster-based permutation was applied to these thresholded *t*-maps and randomization was replicated 5000 times in order to obtain the largest random clusters. The cluster-level significance for the original clusters was then calculated as the percentile of the cluster size compared to the largest random clusters across the 5000 permutations. Additionally, considering the low spatial resolution of EEG/MEG of our source localization method for deeper brain areas (for more details, see Liu et al. 1998; Hauk et al. 2011; Farahibozorg et al. 2018), before conducting cluster-based permutation, the areas highlighted in green in Figure 1*b* were excluded.

### Defining ROIs and Extracting ROI Time Courses for DCM

We defined four candidate semantic hub regions in ATL, IFG, MTG, and AG as the key heteromodal semantic areas proposed in previous literature, in particular with reference to the meta-analytic evidence by Binder et al. (2009) and a review by Pulvermüller (2013). It is worth noting that subdivisions of the temporal cortex in heteromodal semantics are not fully established, and previous studies have considered different numbers of subregions (Binder et al. 2009; Pulvermüller 2013; Lambon Ralph et al. 2016; Jackson et al. 2018). Here, we defined MTG based on the aforementioned meta-analytic evidence, except for the anterior part of the temporal lobe, which was defined as an independent ROI informed by studies of semantic dementia and the hub-and-spokes model of semantics (Patterson et al. 2007; Lambon Ralph et al. 2016). Additionally, we defined Angular Gyrus and parts of the Supramarginal Gyrus (SMG) as one seed labeled “AG.” This seed has been identified as a key semantic area by Binder et al. (2009). Figure 1*c* shows the defined ROIs.

In order to extract each ROI time course, we first identified a vertex within the ROI that showed the highest sensitivity to that ROI. To this aim, we computed cross-talk functions (CTFs) and identified the vertex inside that ROI that showed the largest mean CTF value with other vertices in the ROI, on average across participants (for details of ROI_CTF_ calculations, refer to Liu et al. 1998; Hauk et al. 2011; Farahibozorg et al. 2018). Thereafter, we extracted ROI time courses based on this vertex for each subject-level timeseries that were morphed to fsaverage brain.

### Dynamic Causal Modeling

Our DCM analysis focused on identification of the organization of effective connectivity among the aforementioned candidate hubs: left ATL, IFG, MTG, and AG. The visual word form area (VWFA) in the posterior fusiform gyrus of the left hemisphere was used as the input region.

As the first step, we computed evoked source estimates in the same manner as outlined in Section Source-Reconstructed Evoked Analysis with two exceptions. First, since DCM for Evoked Related Potentials (ERP) requires signed evoked responses (i.e., reflecting the direction of current flow), we here computed source reconstructed ERPs for dipole components perpendicular to the cortical surface based on the aforementioned source estimates with loose orientation constraint. Second, in order to obtain more compatibility with the previous DCM ERP literature (Garrido et al. 2008; Phillips et al. 2015; Chennu et al. 2016), we used a band-pass filter between 1 and 35 Hz. Next, as elaborated earlier, we used CTFs to identify the vertex with the highest sensitivity to each ROI, the time course of which was extracted and utilized in the subsequent analyses.

**Figure 2:**
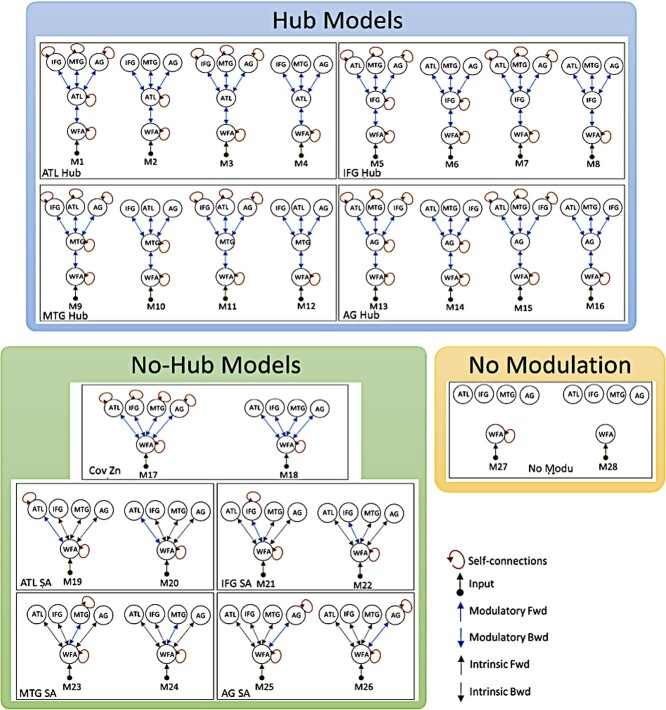
DCM model space delineating grand-families and fine-grained families encompassing 28 models. ATL: Anterior temporal lobe, IFG: Inferior frontal gyrus, MTG: Middle temporal gyrus, AG: Angular gyrus, WFA: Word form area, Cov Zn: Convergence zone, SA: Semantic area.

After extraction of the ROI time courses, we used SPM12 (version r6909) for DCM analysis. The model space, as displayed in Figure 2, comprised 28 models. We defined a hierarchical organization of DCM families in two levels. In the first level of hierarchical comparison, the 28 models were categorized into three “grand” families of hub models, no-hub models, and no-modulation models. Subsequently, in the second level of hierarchical comparison, fine-grained families within the winning grand-family from hierarchy 1 were compared (Figure 2). Models within each fine-grained family spanned different scenarios of self-modulation of the candidate hub areas. It is worth noting that we chose to include the self-modulation of VWFA in all the models. In this way, we can avoid attribution of any possible modulations within this region to the candidate hubs. Finally, we compared single models within the winning fine-grained family in order to examine whether or not one of the models stood out as a conclusive winner.

Each model included evoked responses to both concrete and abstract words, and was fit for each participant separately. Intrinsic connections were assumed to be common between the conditions, while extrinsic connections were used to model condition-induced modulations of the preselected set of connections. Each model was inverted within two time-windows of 0–250 and 0–450 ms, where the former was considered an “early” time-window and the latter was considered a “later” time-window. Because DCM is a dynamical system modeling of signal in response to a perturbation, it requires the data to start at the point of stimulus onset. Thus, both early and late time-windows start from 0 ms (i.e., stimulus onset). Data were reduced to eight spatial modes, and we used the traditional ERP model for DCM inversion (David et al. 2006), with no down-sampling, detrending or Hanning window. Furthermore, considering the lengths of the time windows of the DCM analysis (i.e., 250 and 450 ms), we included modulations of both forward and backward connections in the model (cf., Garrido et al. 2007). This choice was made heuristically and informed by the previous literature where semantic effects have been reported as early as 150 ms (Moseley et al. 2013).

Finally, we used family-level Bayesian Model Selection (BMS) with Fixed Effect Inference (FFX) on the free energy approximation to the model evidence, in order to identify the winning families in each hierarchy of DCM evaluations (Stephan et al. 2007). In the FFX inference, the free energy values were pooled across the three tasks in order to identify the families that provided the highest overall Bayesian evidence. FFX was considered as a suitable approach for the current study because we are studying a homogenous group of healthy young adults, and therefore it is reasonable to assume that the same model applies for all participants. We verified that winning models were not driven by outliers in the free energy.

## Results

### Behavioral Results

We computed reaction times (RTs) and error rates (ERs) for abstract and concrete words for the concreteness decision task from the first experiment and lexical decision task from the second experiment. In the concreteness decision task, we found RTs for abstract versus concrete: 879 ± 118 versus 778 ± 111 ms, ERs: 8.1 ± 5.0% versus 4.5 ± 4.3%. For the lexical decision task, we found RTs for abstract vs concrete: 658 ± 67 versus 664 ± 73 ms and ERs: 4.1 ± 4.8% versus 6.8 ± 3.8%. Note that the semantic target detection task from the second experiment did not provide any behavioral output, considering that participants were only asked to respond to target items that were irrelevant for the main analyses.

### Whole-Cortex Source-Reconstructed Evoked Analysis

We used the contrast of the cortical evoked brain responses to the concrete and abstract words to identify potential hubs based on modulation of their activity. We defined four time-windows of interest covering multiple stages of written word comprehension: 50–150, 150–250, 250–350, and 350–450 ms (which we will subsequently refer to by their central time points, e.g., 100 ms for 50–150 ms). In line with the hub-and-spokes model, we hypothesized the hub activity to be modulated at the earliest stages of semantic processing. As outlined earlier in Materials and Methods, we used EEG/MEG source reconstruction, together with vertex-wise GLM and spatiotemporal cluster-based permutations.

**Figure 3:**
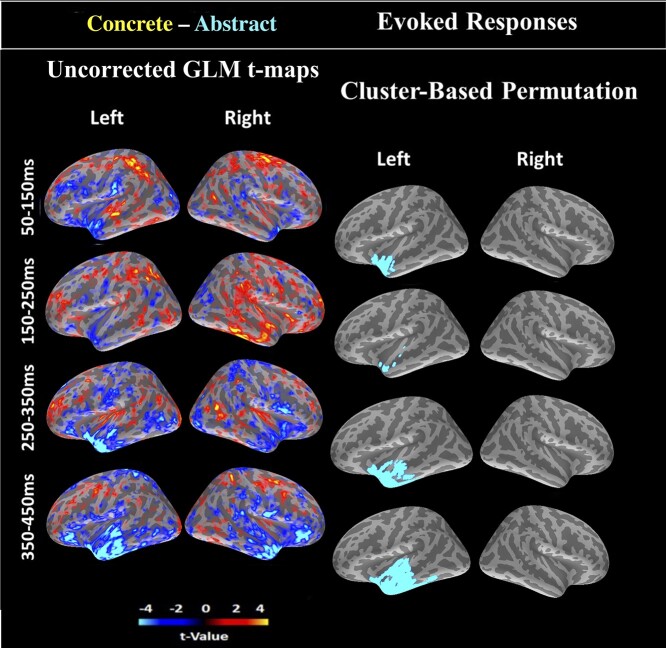
Whole-cortex evoked responses for concrete minus abstract words: Brain activity was averaged within four time windows of 100 ms duration between 50 and 450 ms. LEFT: Uncorrected univariate *t*-statistics from vertex-wise GLM analysis of the main effect of concreteness across three tasks. RIGHT: Significant cluster of spatiotemporal cluster-based permutation tests. Warm colors indicate higher values for concrete words, and cool colors for the abstract words.

When comparing the two word types, computing the main effect of concreteness across all three tasks, we found only one significant spatiotemporal cluster (cluster *P* < 0.05), which was localized to the left ATL (Figure 3) and was modulated between 50 and 450 ms. Throughout all time windows, this cluster showed higher amplitudes for abstract than concrete words. Results of the cluster-based permutation and uncorrected *t*-maps from GLM are shown in Figure 3. In Supplementary Material 3 (Supplementary Figure 3), we show vertex-wise timecourses of the ATL cluster for the contrast of concrete vs abstract, as well as the average (unsigned) timecourse per word category within this cluster. Additionally, in Supplementary Material 4 (Supplementary Figure 5*a*) we show vertex-average timecourses of the ATL cluster (unsigned) for each task separately. No other ROIs showed significant activity differences in whole-cortex evoked analysis.

Next, in order to examine the more fine-grained temporal trajectory of the ATL cluster, and determine the cluster onset, we conducted post-hoc tests within the significant spatial ATL cluster only. In these tests, we first conducted one spatiotemporal cluster-based permutation per time window, with each time window divided into small 10 ms increments: 50:10:150, 150:10:250, 250:10:350, and 350:10:450 ms. We additionally conducted spatial cluster-based permutations on each of the 10 ms time windows separately, with permutations used for correction across ATL voxels, and FDR used for correction across 40 time windows. Results are shown in Supplementary Material 3 (Supplementary Figure 4), where we found 50–150 ms as the earliest modulation time window (with the more fine-grained onset at ~100 ms onwards), and the largest effects were found in 250–350 and 350–450 ms.

### Dynamic Causal Modeling

Having established the effect of concrete vs abstract words on cortical activity, we turned to their effects on cortical connectivity; specifically, between the key heteromodal semantic areas (i.e., candidate hubs) in ATL, IFG, MTG, and AG. For this purpose, we compared a range of network configurations shown in Figure 2 using DCM to identify the model that best explains the evoked responses in those regions. The candidate hub areas were defined based on the previous literature, as outlined earlier in Materials and Methods, and we used VWFA as the input region in our DCM models.

The aim of the DCM analysis was to test: 1) whether this heteromodal network is centered around a hub in the latency ranges 0–250 and 0–450 ms, and 2) if so, which of the four regions act as such a hub. For this purpose, we constructed a hierarchy of families of DCM models, and in each level of comparison compared families within that hierarchy.

#### First Hierarchy: Grand-Family of Hubs Showed the Highest Model Evidence

In this analysis step, we compared three grand families of models (hub, no-hub, and no-modulation families, shown in Figure 2) using BMS. The “hub family” consisted of models 1–16, where either of the candidate hub areas amongst ATL, IFG, MTG, and AG played the role of a single hub that received input from the VWFA and was connected to all other semantic areas in the model space. The “no-hub family” consisted of models 17–26, where in models 17–18, all the candidate hub areas received input directly from the VWFA, and their connections to the input region were modulated by semantic variables, which will resemble a multi-hub or a multiple convergence zones model. In models 19–26, connections of the VWFA to only one of the semantic areas were modulated by the semantic contrast (no further connections to the rest of the network were modulated). Finally, in the “no-modulation” family, models 27–28 had no connection that was modulated by concreteness (differing only in the presence/absence of self-connections in the VWFA).

**Figure 4:**
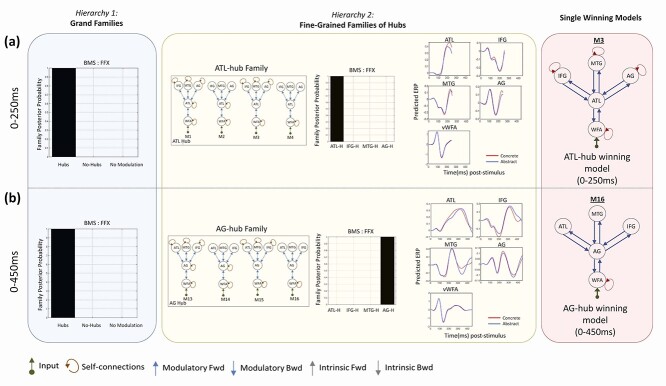
DCM results based on two hierarchies of family comparisons: (*a*) DCM results within 0–250 ms. Hub models and ATL-hub model were identified as winning families of the first and second hierarchy of comparisons, respectively. Model 3 (M3) within the ATL-hub family was identified as a conclusive winner. (*b*) DCM results within 0–450 ms. Hub models and AG-hub model were identified as winning families of the first and second hierarchy of comparisons, respectively. Model 4 (M16) within the AG-hub family was identified as a conclusive winner. BMS: Bayesian model selection, FFX: Fixed-effect inference, ATL-H: ATL-hub, IFG-H: IFG-hub, MTG-H: MTG-hub, AG-H: AG-hub.

Accumulated Bayesian model evidence across the participants from all the tasks identified the hub family as the winning grand family (Figure 4—left panel), within both 0–250 and 0–450 ms post-stimulus windows.

#### Second Hierarchy: ATL and AG Hub Families Showed the Highest Model Evidence

In the next step, in order to find areas that serve as a hub, we compared the fine-grained families within the grand-family of hub models, where a single candidate hub in the intermediate layer of the network linked the input region in the VWFA (via bidirectional connections) to the remainder of the candidate hubs. This grand family consisted of four fine-grained families with an ATL hub, IFG hub, MTG hub, or AG hub. Each of these fine-grained families consisted of four models where the hub received input from the VWFA and established connections to the other nodes of the heteromodal subnetwork, differing only in which self-connections were modulated by word type. BMS results are shown in Figure 4—middle panel. We found that models with ATL as the hub showed the highest posterior probabilities for the 0–250 ms time window, and models with AG as the hub showed the highest posterior probabilities for the 0–450 ms time window (Figure 4—middle panel).

One question that might arise from these results is why does the winning hub family change completely from the earlier to the more prolonged time window (posterior probabilities of winning families > 0.99), and does this imply that ATL’s hub-like behavior completely diminishes over time? To address this, it is worth noting that the posterior probabilities of the winning models relate to the “relative” log model evidence or Bayes factor. Therefore, the winning families with posterior probability ~1.0 show that the accumulated log model evidence across participants and tasks is >150 more than the next best family (Kass and Raftery 1995). This does not imply that the model evidence for the non-winning families is poor; it rather means that the evidence for the winning family is strong enough to prefer it over the other models considered (and therefore does not imply that there is evidence that ATL ceases to function as a hub at later timepoints; only that evidence supports AG as a more likely hub). Relative log model evidence of all the 28 DCM models for each task are shown in Supplementary Material 4 (Supplementary Figure 5).

#### Single Models within ATL and AG Hub Families

Having identified ATL and AG as the most likely hubs within 0–250 and 0–450 ms post-stimulus, respectively, we compared models within each family. Each family comprised four models in which interareal connections were bidirectional and self-modulations were present on the VWFA, but the self-modulation on the remaining ROIs varied across models (for more details, refer to Materials and Methods). Results are shown in Figure 4—right panel. Within the ATL-hub family in the 0–250 ms time window, model 3 in Figure 2 was identified as the conclusive winner, with average free energy estimations for the four models: M1: −710.79, M2: −646.7, M3: −635.78, and M4: −674.31. In this model, all ROIs except the ATL had self-connections that were modulated by word type. Within AG-hub family in 0–450 ms time window, model 16 (i.e., 4th model) was identified as the conclusive winner, with average free energy estimations for the four models as M13: −2076.1, M14: −2121.11, M15: −1992.3, and M16: −1925.4. In this model, none of the ROIs (except VWFA) had self-connections that were modulated. Therefore, in the earlier time window with ATL-hub as the winning family, there was more extensive modulation of within-ROI and cross-ROI connectivity in the single-winning model.

In summary, our DCM results support a single semantic hub model among the heteromodal semantic areas in ATL, IFG, MTG, and AG. These results further reveal a fuller network modulation during earlier compared with the more prolonged time window and uncover ATL as an early hub and AG as a later hub during the course of semantic word processing.

## Discussion

We revealed new aspects of the spatiotemporal cortical network that underlies semantic word processing. For this purpose, we used source-reconstructed electro- and magnetoencephalography data from two experiments and three tasks. Our results provide novel evidence for distinct roles of the anterior temporal lobe (ATL) and angular gyrus (AG) within this network based on two lines of evidence. First, our whole-cortex GLM analysis of evoked brain activity revealed left ATL as the first and only cortical region modulated by word concreteness within 150 ms after stimulus presentation. This persisted into later stages, especially the N400 time window, supporting a central role for the ATL within this network. Second, Dynamic Causal Modeling (DCM) of effective connectivity among the key semantic hub candidates (i.e., ATL, IFG, MTG, and AG) favored “single hub” models in both time windows of investigation (i.e., within 250 and 450 ms post-stimulus), with the left ATL-hub family winning in the earlier time-window, but the AG-hub family winning in the prolonged time-window. Therefore, our results suggest that while both activity and connectivity of ATL are modulated by semantics, especially during earlier stages of semantic information retrieval, AG also supports semantic connectivity especially at later stages.

### Only Left ATL’s Activity Was Modulated by Semantic Variables

Left ATL’s activity modulation within 150 ms post-stimulus provides novel data-driven evidence in support of the key role of this region in semantic processing. This finding is in line with increasing evidence from neuroimaging and electrophysiology literature in recent years based on a variety of tasks and paradigms (Hauk et al. 2012; van Ackeren and Rueschemeyer 2014; Westerlund and Pylkkänen 2014; Jackson et al. 2015; Rice et al. 2015; Zhang and Pylkkänen 2015; Binney et al. 2016; Chen et al. 2016; Lambon Ralph et al. 2016).

We observed the earliest effects in the time window of 50–150 ms post-stimulus, showing ATL activity modulation at ~100 ms onwards. This is slightly earlier than the previously reported ATL effects at ~150 ms (Teige et al. 2018) or 200 ms (Hauk et al. 2012). This finding is plausible in light of the previous research on ATL modulation by semantic composition from 150 to 250 ms onwards (Bemis and Pylkkänen 2011, 2013a, 2013b; Westerlund and Pylkkänen 2014; Zhang and Pylkkänen 2015). For example, Bemis and Pylkkänen (2011) reported ATL sensitivity to minimal composition settings (e.g., red boat) at ~225 ms and Westerlund and Pylkkänen (2014) reported an interaction of semantic composition and specificity in the ATL from 220 ms. As argued by Pylkkänen (2019), for the left ATL to be able to combine two word meanings as of 200 ms, the semantic system must have, in full or in part, retrieved those concepts by that time, which is in line with our finding of ATL modulation from ~100 ms. This early timing can be considered a crucial factor in support of the hub-and-spokes framework, where ATL as a semantic hub is proposed to act as the first link between perceptual and semantic stimulus representations (Rogers et al. 2004; Patterson et al. 2007), thus implying the modulation of the hub region prior to any other semantic areas.

In addition to these early effects, left ATL activation peaked in the established N400 latency range. Generally, we found higher absolute activations for abstract words in all the time windows, likely reflecting higher processing demands on the semantic system, as has been also implicated in previous studies (Binder et al. 2005; Dhond et al. 2007; Lau et al. 2013; Jackson et al. 2015). Our approach to search across cortical vertices and several time windows and combine data from three tasks is unprecedented among EEG/MEG literature, and was specifically chosen to allow for data-driven discovery of any possible contributing regions, and their changes over time, without a-priori restrictions to a few regions of interest. It can thus be expected to improve the generalizability of our results in support of the left ATL as a semantic hub.

### Evidence for a Single Semantic Hub Based on Dynamic Causal Modeling

While early modulation of the left ATL’s activity shows a key role for this region in semantic processing, our DCM results provide key new evidence in favor of a single semantic hub among the heteromodal semantic areas, whose effective connectivity depends on word concreteness. More specifically, we tested models with a single semantic hub against models with no hubs or multiple convergence zones in two different time windows (within 250 and 450 ms). Accumulated Bayesian model evidence across multiple tasks favored models with a single hub as a conclusive winning grand family. Further investigation of the fine-grained families within this grand family revealed ATL-hub as the winning family in an early latency window (0–250 ms), and AG-hub in the more prolonged time-window (0–450 ms). Considering the ATL-hub family winning in the early time window, AG is therefore likely to serve as a hub during later stages (i.e., post 250 ms). Thus, these results suggest that the heteromodal semantic subnetwork is coordinated by a central hub, and suggest that this hub region is dynamically relocated, from ATL to AG, as a semantic processing unfolds in the brain.

Within the ATL-hub winning family, we found a single winning model where all intrinsic and extrinsic connections except for the self-connections of the ATL were modulated by the concreteness contrast, while within AG-hub, the single winning model showed modulation of the extrinsic but not the intrinsic connections of the semantic areas. These results suggest that connectivity within the semantic system is more extensively modulated during the earlier time window of 0–250 ms.

ATL and AG are two brain regions that are frequently implicated in semantic processing, but the division of labor between them in terms of functioning as semantic hubs has remained unresolved to date (Humphreys et al. 2015; Binder 2016; Lambon Ralph et al. 2016). Our results draw a distinction between their roles based on: 1) activity versus connectivity and 2) earlier versus later modulation. This distinction can be examined from two angles:

First, from a methodological perspective, one may ask why DCM for evoked responses identifies AG as a hub during later stages while its evoked activity shows no modulation. This can be explained in light of how the winning models are determined: in DCM, the winning models denote architectures that can minimize the differences between the observed and predicted time courses, while penalizing for model complexity. As a result, the winning model is expected to be overall best at explaining the temporal trajectories of each ROI for each word category, as well as explaining the differences between the word categories. Therefore, even though AG activity per se is not modulated by semantics, the models where this region coordinates effective connectivity within the semantic network provide the highest Bayesian evidence.

Second, from a cognitive perspective, we must ask why the hub region of effective connectivity changes over time from ATL to AG even though the modulation of neural activity remains localized to the ATL? The inferior parietal lobe (IPL) in general and AG in particular are well known to be involved in higher cognitive function, not only for semantics (Binder et al. 2009; Bonner et al. 2013; Hartwigsen et al. 2016; Kuhnke et al. 2020; Soto et al. 2020), but also for broader cognition such as episodic memory and executive functions for complex task demands (Humphreys et al. 2015; Humphreys and Lambon Ralph 2015; Bonnici et al. 2016; Tibon et al. 2019). Additionally, as a key node of the brain’s default mode network, IPL has been suggested as a multi-purpose hub in the brain (Buckner et al. 2009; Seghier 2012; Raichle 2015), and has been shown to be affected by brain diseases such as Alzheimer’s and dementia (Buckner et al. 2008; Spreng et al. 2010). Put together with this past evidence, our findings thus indicate that AG is likely to accumulate information from the semantic system to act as a bridge between different memory and cognitive brain systems at later processing stages. In contrast, ATL as the central semantic hub may initiate and coordinate activity within the semantic system through cortico-cortical connections from the earliest stages of processing.

### Caveats

The choice of connectivity metrics for EEG/MEG analysis is still challenging. Here we used DCM to be able to explicitly test some of the predictions of the hub-and-spokes model against alternative theories. DCM is arguably the sole available method for full modeling of the evoked responses to experimental manipulations based on explicit network models. Even so, the model evidence can only find the most likely model among those tested, so it cannot determine whether the winning model is in fact the true model (Litvak et al. 2019). In particular, inspired by the previous literature (Binder et al. 2009; Martin et al. 2014; Lambon Ralph et al. 2016), we here only focused on different scenarios spanning one-layer networks with single or multiple parallel semantic areas or two-layer models with a single hub in the intermediate layer. Nevertheless, current findings do not obviate the possibility of more complex heteromodal semantic networks with different permutations of semantic areas in three or more network layers, potentially involving multiple hubs. Moreover, DCM makes several strong assumptions (Stephan et al. 2010; Friston et al. 2019), the robustness of which need to be validated in future studies using other datasets, including more direct electrical recordings in humans and animals. Our results are therefore presented as a significant step forward towards the delineation of time-resolved semantic network connectivity which will open new doors for future investigations.

We utilized a word concreteness contrast across multiple visual word recognition paradigms which has been successfully and frequently used as the sole task in previous fMRI (Binder et al. 2005) and EEG/MEG (Dhond et al. 2007) studies, and can be assumed to modulate the heteromodal semantic regions, in particular, candidate hubs in the ATL, IFG, MTG, and AG. Even though we combined evidence across multiple tasks and experiments to minimize task-specific effects, some of our findings might still be specific to the contrast of concrete and abstract words. Therefore, an important next step from our study is to tackle the time-resolved semantic network for more general and more specific semantic contrasts, as well as different task settings such as auditory word presentations or nonverbal visual inputs. Furthermore, plausible reorganizations of the semantic network due to different tasks is another important question that remains to be tackled in future studies.

## Conclusion

Our study provides novel insights into the dynamic brain networks underlying semantic word processing that could not have been provided using metabolic neuroimaging or neuropsychological methods. Analyzing the time course of activation and connectivity in this study has allowed the integration of previously distinct approaches to neural semantic networks. In particular, we could shed new light on the central role of the ATL for early semantic processing as previously indicated by both patient and imaging work (Patterson et al. 2007; Lambon Ralph et al. 2016), as well as on AG’s role in the accumulation of information from the semantic system for integration with broader memory and cognitive processes (Binder et al. 2009; Humphreys and Lambon Ralph 2015; Bonnici et al. 2016).

## Supplementary Material

supplementary_materials_bhab501

## Data Availability

The code used to analyze the data in this study is available from the following repository: https://github.com/rezvanfarahi/SemNet. The datasets used in the current study are not publicly available due to the sensitivity of the data but can be made available upon request from the corresponding author.

## Notes

We would like to thank Professor Karalyn Patterson for helpful discussions on earlier versions of this work, Dr Rebecca Jackson for feedback on the manuscript, and Drs Elisa Cooper, Yuanyuan Chen, and Gemma Evans for their help with data collection. *Conflicts of Interest*: The authors declare no conflicts of interest.
