# Supplementary material for: Distinct roles for the anterior temporal lobe and angular gyrus in the spatiotemporal cortical semantic network
Source: Cereb Cortex. 2022 Jan 30;32(20):4549–64. doi: 10.1093/cercor/bhab501 (PMC9574238; doi:10.1093/cercor/bhab501)
Supplement: supplementary_materials_bhab501 [file supplementary_materials_bhab501.pdf]

Distinct roles for the Anterior Temporal Lobe and Angular Gyrus in the spatio-temporal cortical semantic network

Farahibozorg SR, Henson R, Woollams AM, & Hauk O

Supplementary Materials

S 1 Behavioural word rating study in experiment 2

This behavioural study was conducted as a pre-EEG/MEG experiment in order to provide materials for the main EEG/MEG study. The purpose of this experiment was to provide lists of concrete and abstract word categories (50 words per category).

S.1.1 Participants

41 healthy native adult English speakers (aged 18-75) participated in the study, but one subject was removed due to inaccuracies in following the instructions. Hence, 40 participants (age 38.3±17.9, 25 female) entered the final analysis. All participants had a normal or corrected-to-normal vision with no reported history of neuropsychological disorders or dyslexia. The experiment was approved by the Cambridge Psychology Research Ethics Committee and volunteers were paid for their time and effort.

S.1.2 Stimuli

A set of 586 words was collected for rating and subjects were asked to make judgements about concreteness, visual, auditory and hand-action attributes of each word (details below in S.1.3). We initially selected the words subjectively (checked by two authors) such that they were associated with at least one of the attributes of interest in order to ensure a suitable preliminary list. However, the final evaluations were solely based on the rating study. In order to maintain subjects' efficiency and avoid a prolonged experiment, participants were divided into two groups of 20 and each group performed the rating for half of the word list.

S.1.3 Procedure

The word rating experiment consisted of four blocks with an overall duration of approximately 90 minutes including three short breaks between the blocks. In each block, subjects rated 293 words in response to the following four questions focused on concreteness, visual-relatedness, auditory-relatedness and hand-action relatedness of the concepts. Participants were given a maximum of 8 seconds to rate each word and were asked to rate on a scale of 1 to 7 with 1 indicating a low rating and 7 corresponding to a high rating. The experiment was implemented in PsychoPy (version 1.82.01) (Peirce 2007) and details of the questions were:

1- How concrete is the following word?

Concrete words refer to persons, places and things that can be seen, heard, touched, smelled, tasted or acted upon, and abstract words refer to the concepts that cannot be experienced by your senses. Concrete words should receive high ratings and abstract words should receive low ratings. Please rate on the scale of 1 to 7 with 1 indicating highly abstract and 7 indicating highly concrete.

2- Does the following word immediately and vividly remind you of something you can hear?

If a word meaning is strongly associated with auditory properties, including but not restricted to sounds, music and voices, you should rate it highly. Otherwise, it should be given a low rating. Please rate on the scale of 1 to 7 with 1 indicating not at all and 7 indicating extremely.

3- Does the following word immediately and vividly remind you of an action that you can perform with your hands?

Only if a word is strongly associated with hand actions, you should rate it highly. If it is associated with actions of other body parts or not associated with actions at all, it should be given a low rating. Please rate on the scale of 1 to 7 with 1 indicating not at all and 7 indicating extremely.

4- Does the following word immediately and vividly remind you of features that you can visually perceive?

If a word meaning is strongly associated with visual properties, including but not restricted to shape, form and colour, you should rate it highly. If you do not think that the word is associated with visual features at all, it should be given a low rating. Please rate on the scale of 1 to 7 with 1 indicating not at all and 7 indicating extremely.

The response times to questions 1-4 were 1.99 ± 0.41, 1.67 ± 0.26, 1.54 ± 0.24 and 1.63 ± 0.25 seconds, respectively. In order to find 50 words per category, we used Match software (Van Casteren and Davis 2007) and identified concrete and abstract words that were matched with respect to a group of psycholinguistic variables elaborated in section 2.2. More specifically, based on questions 2, 3, 4 above, we first found 50 matched concrete words from each of the visual, auditory and hand sub-categories, which were then pooled and 50 of those were matched to 50 abstract words.

S 2 Supplement to Methods section 2.4: sensor-space summaries

In Methods Section 2.4 we explained details of sensor-space analysis, up to extraction of Evoked-related activity, which is used as input to the subsequent source localisation and connectivity investigations. Here in FIGURE S 1 and FIGURE S 2 we show a summary of sensor-space results. Note that no statistical comparisons are conducted in sensor space throughout this study.

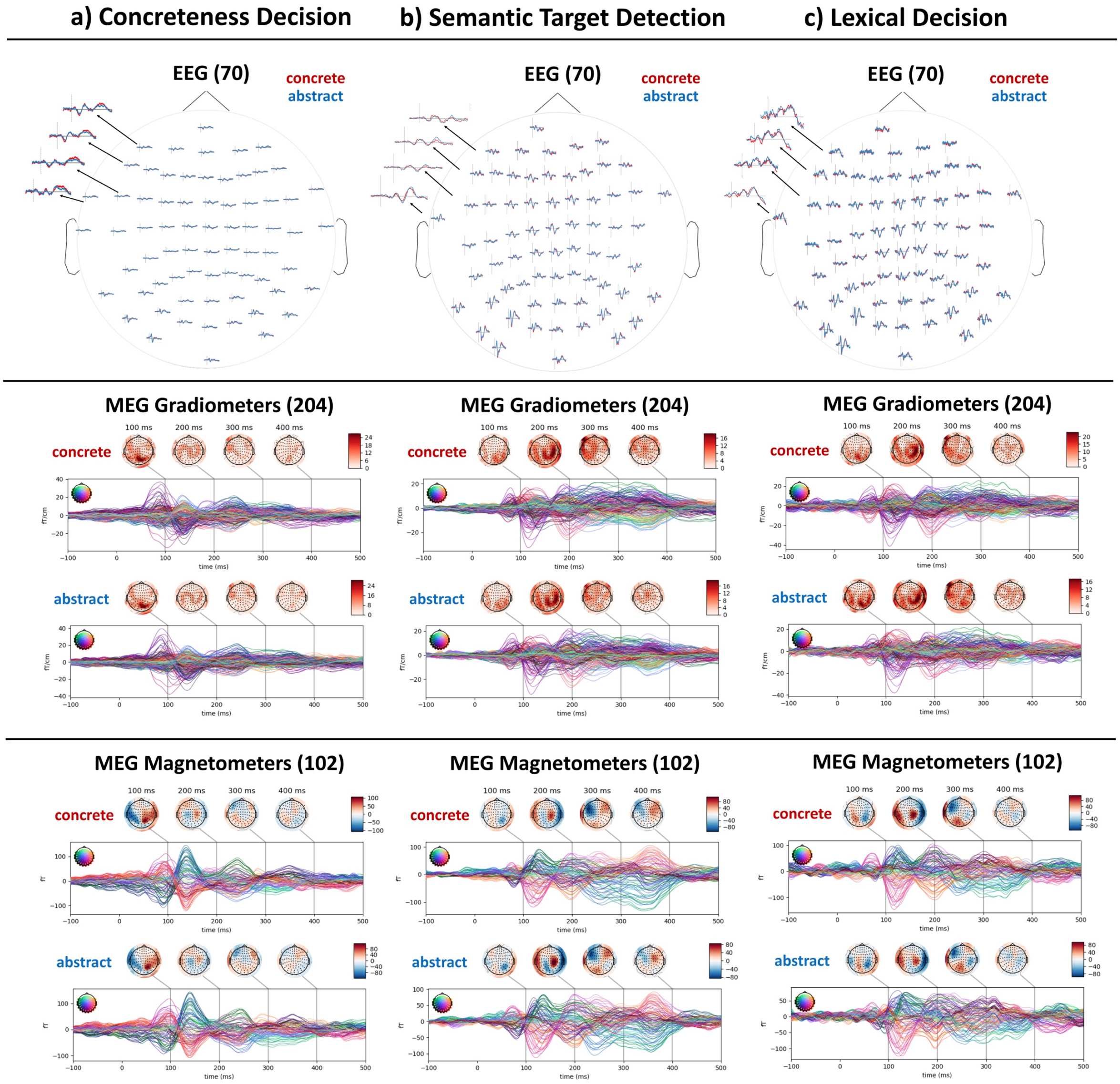

Figure S 1 Evoked brain activity in response to concrete and abstract words per sensor-type and per-task in sensor space. Results are averaged across subjects. a) Concreteness decision task; b) semantic target detection task; c) lexical decision task. Top: EEG results, middle: MEG Gradiometers and bottom: MEG Magnetometers. Numbers in parentheses (70), (204), (102) denote number of sensors.

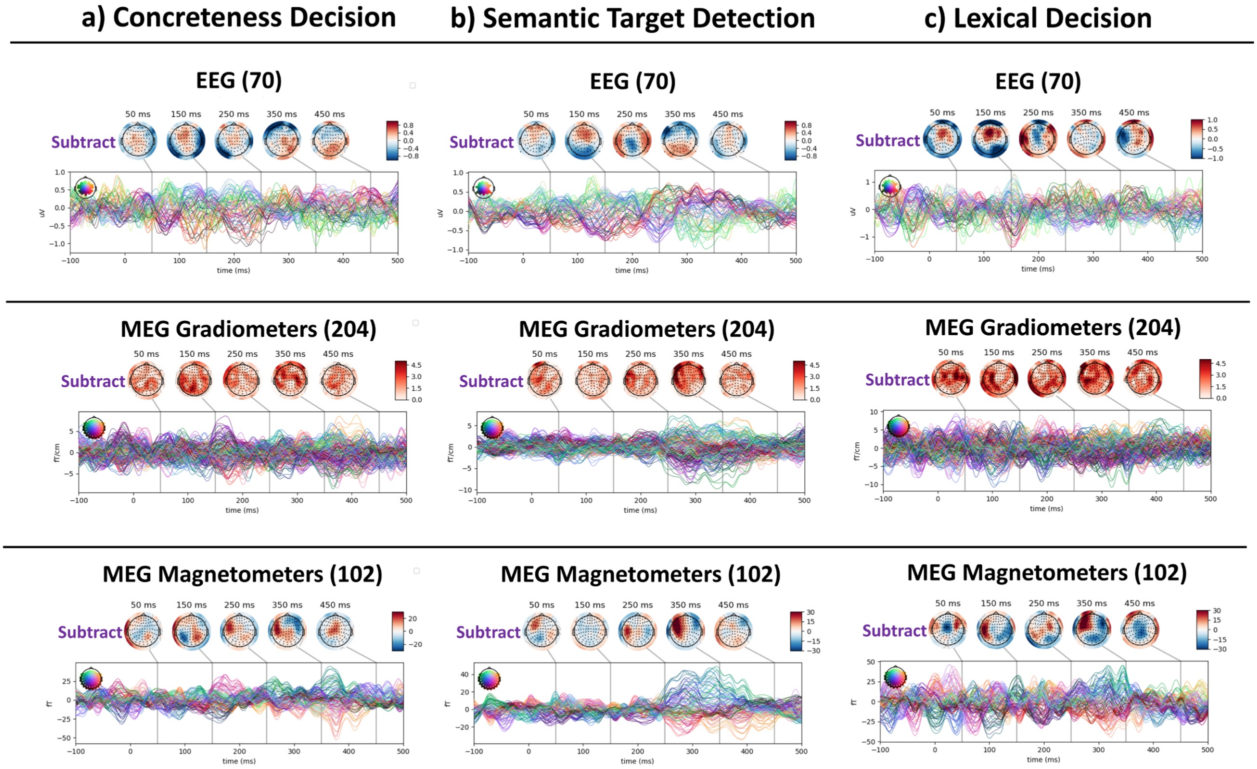

Figure S 2 Evoked brain responses to the contrast of concrete and abstract words (concrete minus abstract) per sensor-type and per-task in sensor space. Results are averaged across subjects. a) Concreteness decision task; b) semantic target detection task; c) lexical decision task. Top: EEG results, middle: MEG Gradiometers and bottom: MEG Magnetometers. Numbers in parentheses (70), (204), (102) denote number of sensors.

S 3 Supplement to Results section 3.2: ATL cluster

In section 3.2, we found that in a whole-cortex spatiotemporal clustering analysis, a cluster in the left ATL was the only region modulated by the contrast of concrete and abstract words between 50-450ms. Here we show vertex-wise and average timecourse of this cluster, and conduct a post-hoc analysis to determine the cluster onset.

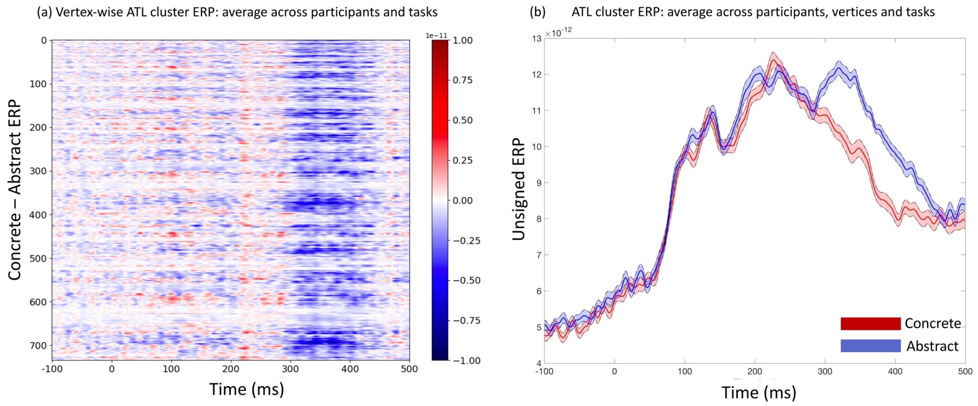

Figure S 3 Timecourse of the ATL cluster found in Figure 3. a) vertex-wise timecourse for the contrast of concrete (red) minus abstract (blue). Data is averaged across participants and tasks. b) Average timecourse (across vertices) of ATL for concrete and abstract words. Averages (solid-line curves) and standard errors (shaded curves) are calculated across participants and tasks.

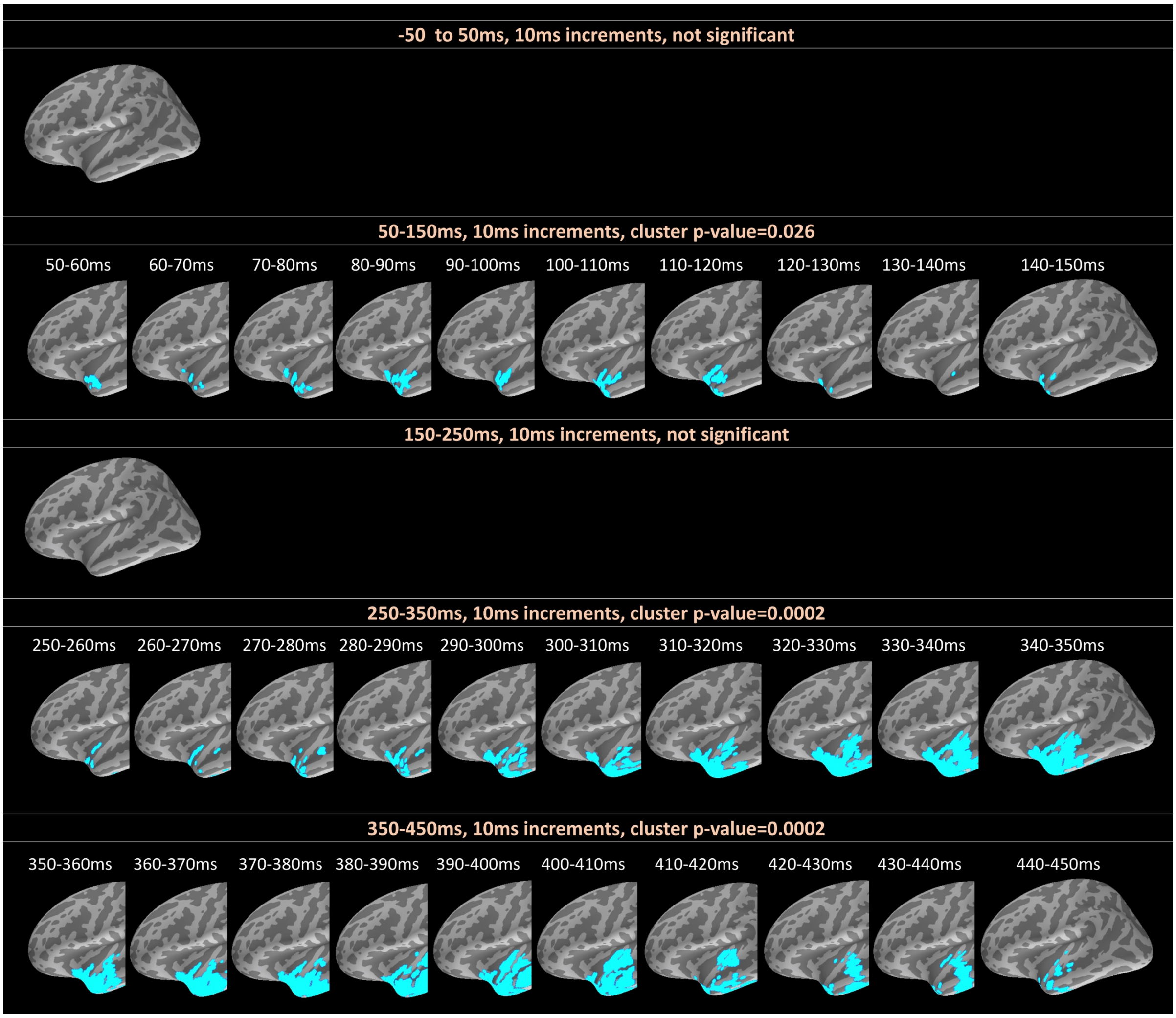

Figure S 4 Post-hoc analyses to examine the temporal trajectory of the ATL cluster reported in 3.2. Here we ran spatio-temporal cluster-based permutations separately for each time window, and masked within ATL cluster only. Here each time window of 100ms is divided into fine-grained 10ms increments. Results are spatio-temporally corrected using permutations within each time window, and further FDR corrected for multiple comparisons across the 5 time windows. Note that here we include an additional time-window of -50 to 50ms to determine the cluster onset more accurately. The first significant cluster was identified in 50-150ms time window, and the largest effects appeared in 250-350 and 350-450ms time windows. We additionally conducted spatial cluster-based permutations separately for each 10ms time-window spanning 50-450ms and FDR-corrected results ($p<0.05$) across 40 time windows to determine the cluster onset more accurately, the following time windows were identified as being significant: 80-90ms, 90-100ms, 100-110ms, 110-120ms, 160-170ms, 170-180ms, 180-190ms, 280-290ms, 290-300ms, 300-310ms, 310-320ms, 320-330ms, 330-340ms, 340-350ms, 350-360ms, 360-370ms, 370-380ms, 380-390ms, 390-400ms, 400-410ms, 410-420ms, 420-430ms, 430-440ms.

S 4 Supplement to Result sections 3.2 and 3.3: per task results

Throughout the main results section, we have reported consensus patterns across three tasks: concreteness decision, lexical decision and semantic target detection. Here in FIGURE S 5, we present a summary of per-task results. However, please note that no statistical analyses are conducted for each task separately.

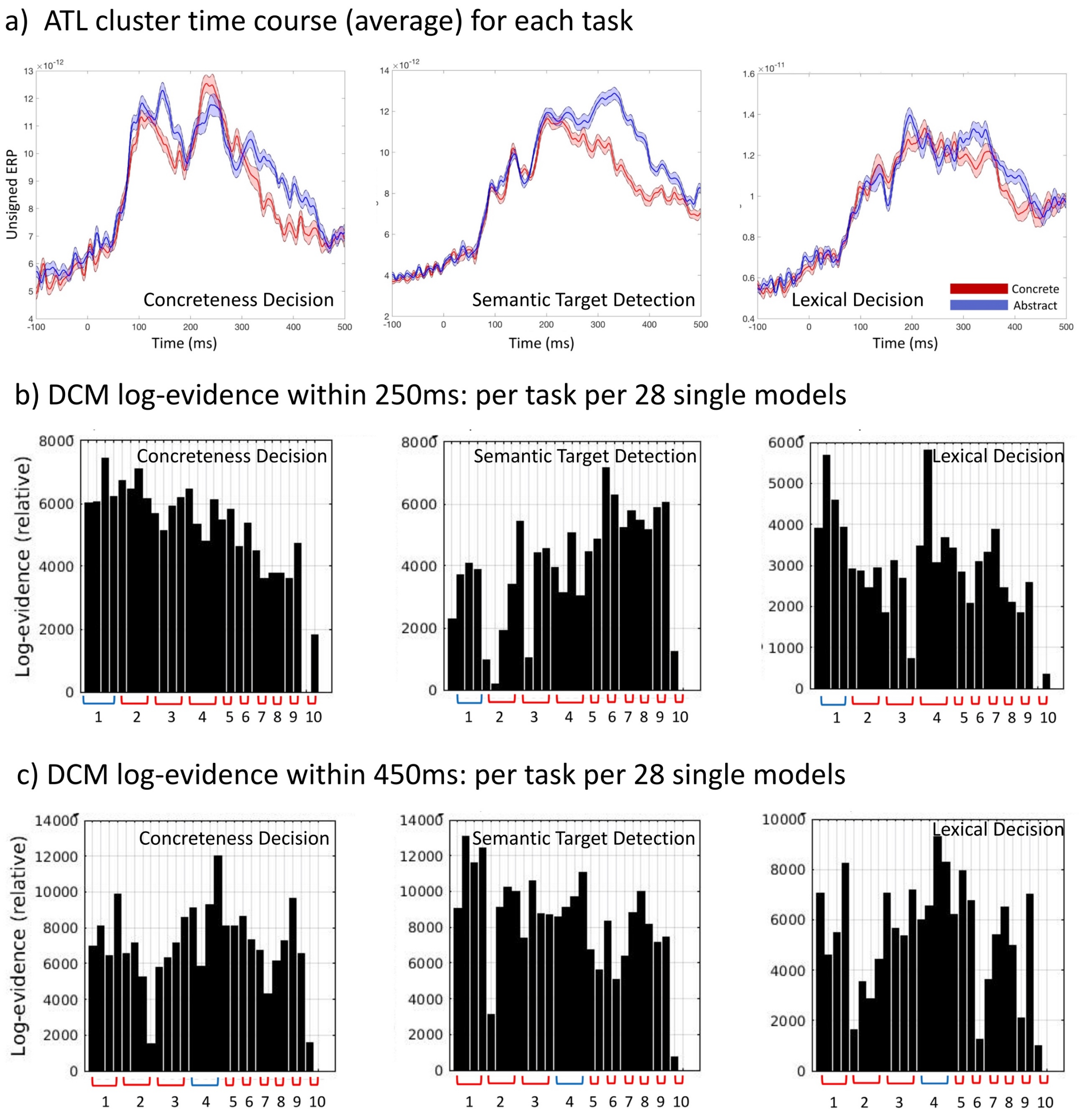

Figure S 5 Per-task results. a) average source localised Evoked activity for concrete and abstract words, for ATL cluster identified in Figure 3; b) Log model evidence (relative) for 28 DCM models illustrated in Figure 2 within 250ms; c) Log model evidence (relative) for 28 DCM models illustrated in Figure 2 within 450ms. Red and blue brackets illustrate DCM families, with blue denoting winning families (i.e., ATL-hub family within 250ms and AG-hub family within 450ms).
